# Numerical Study of Particle Separation through Integrated Multi-Stage Surface Acoustic Waves and Modulated Driving Signals

**DOI:** 10.3390/s23052771

**Published:** 2023-03-03

**Authors:** Yingqi Jiang, Jin Chen, Weipeng Xuan, Yuhao Liang, Xiwei Huang, Zhen Cao, Lingling Sun, Shurong Dong, Jikui Luo

**Affiliations:** 1 Ministry of Education Key Laboratory of RF Circuits and Systems, College of Electronic & Information, Hangzhou Dianzi University, Hangzhou 310018, China; 2 Zhejiang Key Laboratory of Large-Scale Integrated Circuit Design, Hangzhou Dianzi University, Hangzhou 310018, China; 3 Key Laboratory of Advanced Micro/Nano Electronics Devices & Smart Systems of Zhejiang, College of Information Science & Electronic Engineering, Zhejiang University, Hangzhou 310027, China; 4 International Joint Innovation Center, Zhejiang University, Haining 314400, China

**Keywords:** particle sorting, standing surface acoustic waves, multi-stage sorting

## Abstract

The manipulation of biomedical particles, such as separating circulating tumor cells from blood, based on standing surface acoustic wave (SSAW) has been widely used due to its advantages of label-free approaches and good biocompatibility. However, most of the existing SSAW-based separation technologies are dedicated to isolate bioparticles in only two different sizes. It is still challenging to fractionate various particles in more than two different sizes with high efficiency and accuracy. In this work, to tackle the problems of low efficiency for multiple cell particle separation, integrated multi-stage SSAW devices with different wavelengths driven by modulated signals were designed and studied. A three-dimensional microfluidic device model was proposed and analyzed using the finite element method (FEM). In addition, the effect of the slanted angle, acoustic pressure, and the resonant frequency of the SAW device on the particle separation were systemically studied. From the theoretical results, the separation efficiency of three different size particles based on the multi-stage SSAW devices reached 99%, which was significantly improved compared with conventional single-stage SSAW devices.

## 1. Introduction

Biomedical and healthcare diagnostic applications, such as circulating tumor cell (CTC) detection, antibiotic resistance investigation, and medication screening, rely heavily on micro-particle or cell separation and detection by point-of-care (POC) devices [1,2]. Based on different physical properties of particles including size, density, and deformability, a wide variety of microfluidic separation techniques have been developed, which can be divided into active or passive methods. Active separation techniques move particles by applying a force inherited from acoustic [3,4], magnetic [5,6], dielectrophoretic [7], or optical [8,9] fields. Recently, surface acoustic wave (SAW)-based particle sorting approaches have drawn increasing attention due to their paramount advantage of easy implementation and good biocompatibility [10,11,12]. In general, an acoustofluidic-based particle separation technique combines acoustic streaming and acoustic radiation force (ARF) to persistently control particles across a broad size range [3]. Through the vibration of the piezoelectric layer, the acoustic energy could be efficiently coupled to the liquid and accurately manipulate fluids and particles. 

Much work has been devoted to the acoustic wave-based microfluidic device. Early studies mainly focused on two-dimensional numerical modelling [13]. Liu et al. [14] reported on the influence of the input Radio Frequency (RF) signal on particle separation. Shamloo et al. [15] utilized two-dimensional simulations of separation, demonstrating the effect of trapezoidal microfluidic channels on particle separation. Tao et al. [16] proved that pre-focusing would impose an impact on the separation efficiency of particles. SSAW has also been used in other studies for cell sorting in microfluidic devices [17,18,19,20]. Most studies have used the SAW device to separate two different size particles/cells, or to separate two particles of the same size but different materials. The separation of three or more particles at the same time is rarely studied. Wu et al. [21] employed two SAW devices to separate particles in two steps. In each step, particles of different sizes were removed from the sheath flow. 

In this work, the theoretical model of a microfluidic system based on an SSAW device for particle separation was systematically explored using the finite element method (FEM). The 3D model microfluidic device was set up and analyzed. The 128° Y-cut LiNbO_3_ was selected as the piezoelectric substrate. First, the separation characteristics of particles in three different diameters using a single-stage SAW device were analyzed. Based on the results, we proposed a multi-stage SAW device driven by a modulated signal composed of two frequencies in programmable amplitude. The separation efficiency of the device was greatly improved. 

## 2. Numerical Analysis Model

### 2.1. Basic Principles of Acoustic Field and Particle Motion

The mass and momentum balance laws governing the motion of a linear viscous compressible fluid are [22,23]

(1) $$\frac{\partial \rho }{\partial t}+\nabla \cdot \left(\rho v\right)=0$$

and

(2) $$\rho \frac{\partial v}{\partial t}+\rho \left(v\cdot \nabla \right)v=-\nabla p+\mu \nabla^{2}v+\left(\mu_{b}+\frac{1}{3}\mu \right)\nabla \left(\nabla \cdot v\right)$$

where ρ is the mass density, v is the fluid velocity, p is the fluid pressure, and μ and $\mu_{b}$ are the shear viscosity and bulk viscosity, respectively. Here, it is assumed that the fields ρ, p and v are understood to be in Eulerian form [24]; they represent a function of time *t* and space position *r* in a fixed volume. Additionally, a constitutive relation between pressure and density is required to characterize fluid motion. We consider p and ρ to have a linear relationship:

(3) $$p=c_{0}^{2}\rho$$

where *c*_0_ represents the speed of sound in the fluid. Combining Equations (1)–(3) with proper boundary conditions, the system is fully determined.

The acoustic pressure caused by the acoustic wave is a harmonic component, and the harmonic scattering acoustic pressure in the fluid is controlled by the Helmholtz wave equation [25,26]

(4) $$\nabla^{2}p_{a}+\frac{\omega^{2}}{c_{0}^{2}}p_{a}=0$$

where *p_a_* is the acoustic pressure and ω is the angular frequency.

In the microfluidics of the SAW driving system, particles within the SSAW field in the micro-channel encounter four main forces, i.e., gravity (*F*^G^), buoyancy (*F*^B^), acoustic radiation force ($F^{\mathrm{rad}}$), and Stock’s drag force ($F^{\mathrm{drag}}$). For particles suspended in the fluid, gravity and buoyancy are in equilibrium and can be neglected. Thus, the particles moving in a fluid and in the SAW area will be affected by two forces: acoustic radiation force due to the scattering of the acoustic wave and Stokes drag force due to the fluid streaming. 

The time-average acoustic radiation force $F^{\mathrm{rad}}$ on a single spherical particle in a viscous fluid is expressed by [27]

(5) Frad=−πa3[2κ03Re[f1 *p1 *∇p1]−ρ0Re[f2 *v1 *⋅∇v1]]

where a is the radius, $\rho_{0}$ is the density of the particle, and $\kappa_{0}=1/\left(\rho_{0}c_{0}^{2}\right)$ is the compressibility of the fluid. According to the derivation of the literature, here $f_{1}$ and $f_{2}$ are

(6) $$f_{1}\left(\tilde{\kappa }\right)=1-\tilde{\kappa }\;and\;\tilde{\kappa }=\frac{\kappa_{p}}{\kappa_{0}}$$

(7) $$f_{2}\left(\tilde{\rho },\tilde{\delta }\right)=\frac{2\left[1-\Gamma \left(\tilde{\delta }\right)\right]\left(\tilde{\rho }-1\right)}{2\tilde{\rho }+1-3\Gamma \left(\tilde{\delta }\right)}\;and\;\tilde{\rho }=\frac{\rho_{p}}{\rho_{0}}$$

(8) $$\Gamma \left(\tilde{\delta }\right)=-\frac{3}{2}\left[1+i\left(1+\tilde{\delta }\right)\right]\tilde{\delta }\;and\;\tilde{\delta }=\frac{\delta }{a}$$

Particle compressibility is $\kappa_{p}$, particle density is $\rho_{p}\;$, and δ are viscous boundary layer thicknesses. The acoustic energy density is a special case of $E_{\mathrm{ac}}$ for horizontal pressure half-wave resonance, $p_{1}=p_{a}\sin\left(qy\right)$, with channel width w and wavenumber q=π/w. The expression for the radiation force can be simplified to

(9) $$F_{1}^{\mathrm{rad}}=4\pi \Phi \left(\tilde{\kappa },\tilde{\rho },\tilde{\delta }\right)a^{3}qE_{\mathrm{ac}}\sin\left(2qy\right)$$

(10) Φ(κ˜,ρ˜,δ˜)=13f1(κ˜)+12Re[f2(ρ˜,δ˜)]

where Φ is the acoustic contrast factor.

The common expression for the time-averaged Stokes drag force $F^{\mathrm{drag}}$ on a sphere of radius a moving with velocity u in a fluid possessing the streaming velocity $\langle v_{2}\rangle$ is given as [28]

(11) $$F^{\mathrm{drag}}=6\pi \eta a\left(\langle v_{2}\rangle -u\right)$$

where η and a are the liquid viscosity and the radius of a spherical particle, respectively. Newton’s second law is then used to forecast how the bead will move.

(12) $$m_{p}\frac{dv_{p}}{dt}=F^{\mathrm{rad}}+F^{\mathrm{drag}}$$

where $m_{p}$ denotes the bead’s mass. Since the typical time of acceleration is short in relation to the time scale of the particle motion, the inertia of the bead can often be ignored in acoustofluidics situations [29].

### 2.2. Device Structure

**Figure 1:**
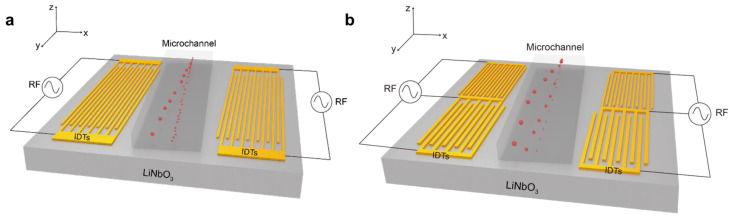
Schematic diagrams of a microfluidic sorting device with single-stage IDT (**a**) and multi-stage IDT (**b**).

The schematic of the SSAW particle separation device is shown in Figure 1. Figure 1a depicts a traditional single-stage SAW device while Figure 1b is the multi-stage SAW device proposed in this work. A pair of identical interdigital transducers (IDTs) are coated on a 128^o^Y-cut LiNbO_3_ piezoelectric substrate. A polydimethylsiloxane (PDMS) microfluidic channel is bonded over the surface of the substrate. To shift the particles in the lateral direction of more than 1/4 wavelength, the channel and the IDT were assembled at an inclined angle [30]. When RF signals are applied to IDTs, the substrate will vibrate due to the inverse piezoelectric effect, producing a propagation of Rayleigh SAWs on the substrate. The acoustic waves travel along the surface and are dissipated into the fluid, creating a distribution of acoustic pressure nodes in the fluid. The acoustic pressure in the fluid causes the particles to be subjected to acoustic radiation forces. As the magnitude of the acoustic radiation force is influenced by the particle’s radius, it will cause the particles to deviate from their original trajectory and move towards the acoustic pressure node. In this work, a simplified but effective 3D model was established, and COMSOL 6.0 software was used to realize the numerical analysis.

### 2.3. Material and Model Parameters

**Figure 2:**
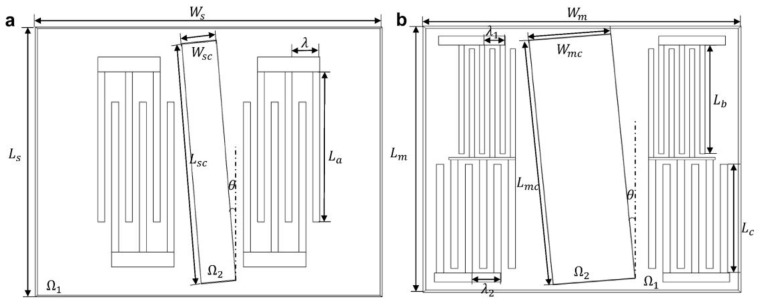
Diagram of the microfluidic particle sorting device with a pair of IDTs (**a**) and multiple pairs of IDTs (**b**).

The numerical analysis was performed on a Windows 10 computer with 64 GB RAM and a 24-core Intel(R) Xeon(R) CPU E5-2650 v4@2.20 GHz CPU. The models proposed are shown in Figure 2, which will be investigated in detail in the following work. One is the traditional single-stage SAW device and the other is a multi-stage SAW device. The part, $\Omega_{1}$, is the 128^o^ Y-cut LiNbO_3_ piezoelectric substrate and aluminum IDTs. For simplification, three pairs of IDTs were used in this simulation. The part, $\Omega_{2}$, represents the microfluidic channel. The channel was rotated counterclockwise by an angle of θ. The angle θ in the figure is 5^o^. The specific parameters of the model are in Table 1.

The materials used in this work are as follows. The material of the fluid in the channel is water. The substrate is LiNbO_3_, and the IDT is aluminum. The particle material is polystyrene. They are shown in Table 2 [31,32,33,34].

### 2.4. Boundary Conditions and Numerical Implementation Process 

For the boundary conditions used in this work, the PML (Perfect Match Layer) boundary was set on the left and right, the back and front surfaces of the LiNbO_3_. For the substrate’s bottom surface, the fixed boundary condition was also set. Half of the IDTs were set as ground, and the RF voltage was applied to the left IDTs. The acoustic pressure generated in the channel could be changed by adjusting the voltage amplitude applied to the IDTs. 

In the area of surface acoustic wave generation, we use two modules, i.e., “solid mechanics” and “electrostatic”, which are coupled by a multi-physics field “piezoelectric effect”. The fluid velocity in the microfluidic channel is calculated using the “laminar flow” module, the sidewalls are set to a non-slip boundary condition, the input is set as a fully developed flow condition, and the output is set as atmospheric pressure. To determine the first-order acoustic pressure and velocity distribution in the channel, the “pressure acoustics, frequency domain (acpr)” module is employed. The impedance boundary condition is used to set the left and right-side walls of the channel as PDMS with impedance $\rho_{PDMS}*c_{PDMS}$ and the surface of inlet and outlet of the channel as water with impedance $\rho_{water}*c_{water}$. The particle trajectory is solved using the “Fluid Flow Particle Tracking” module, and the middle of the inlet of the channel entrance was set as the particle release region. In addition, the diameter and density properties of the particles were set. The particle motion was studied under the influence of acoustic radiation and Stokes traction. A multi-physics “acoustic–structural boundary” is used to couple the solid mechanics and the pressure–acoustic interfaces to transmit the surface acoustic waves into the fluid channel. The multi-physics field “fluid–particle interaction” couples the laminar flow with the fluid flow particle tracking interface, allowing the particles to flow within the channel.

### 2.5. Mesh Convergence

**Figure 3:**
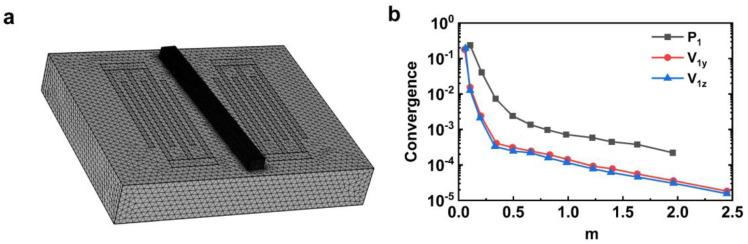
Mesh convergence analysis. (**a**) The computational mesh of the model. (**b**) The relative convergence value under variable mesh size, which is dependent on *m*.

Different mesh sizes will result in different simulation results and simulation times, thus reliable mesh method is necessary. In all simulation models, the tetrahedral mesh grid was used. The mesh size is controlled by the parameter factor m. In all of the models, the maximum mesh size was limited to d/m in the substrate region and d/2∗m in the fluid region, where d is a predefined parameter. λ is the wavelength of the SAW, and d=λ/8 is set for the calculation. Figure 3a shows the grid used throughout the model, which consists of 6.1 × 10^5^ cells. 

The mesh convergence study is carried out by looking at the convergence parameter C(g), which is represented as [35]

(13) $$C\left(g\right)=\sqrt{\frac{\int^{\;}\;{\left(g-g_{\mathrm{ref}}\right)}^{2}\;dy\;dz}{\int^{\;}\;{\left(g_{\mathrm{ref}}\right)}^{2}\;dy\;dz}}$$

where $g_{\mathrm{ref}}$ is the reference solution obtained after grid division when m= 3. The convergence function C in Figure 3b is drawn as a function of m. All variables begin to converge fully when m reaches 0.5, and the convergence results are satisfactory when m= 1.5. For the rest of the work, the grid with the parameter factor of m= 1.5 is used.

## 3. Results and Discussion

### 3.1. Acoustic Pressure Generated by SAW Device

**Figure 4:**
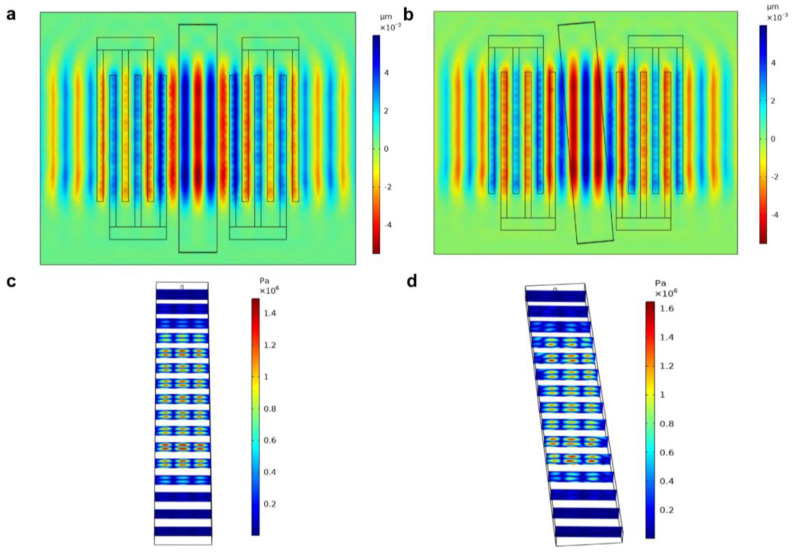
The displacement generated on the substrate surface when the channel was placed in parallel (**a**) and placed with an angle (**b**) to the IDT. The acoustic pressure distribution in the channel when the channel was placed in parallel (**c**) and placed with an angle (**d**) to the IDT.

The Rayleigh wave vibration from the substrate caused the acoustic pressure produced in the microfluidic channel. The displacement on the top surface is shown in Figure 4a,b. According to the previous study [36], if the channel is parallel to the IDT, the maximum displacement of the particle in the channel is in the 1/4 wavelength. Therefore, to increase the lateral displacement, a slanted angle θ between the channel and the IDT was utilized, and the generated acoustic pressure nodal line forms the angle θ with the flow direction. The generated acoustic pressure in the slanted channel is shown in Figure 4d, which shows how the acoustic pressure distribution varies with locations in the channel. 

### 3.2. The Particle Tracing 

**Figure 5:**
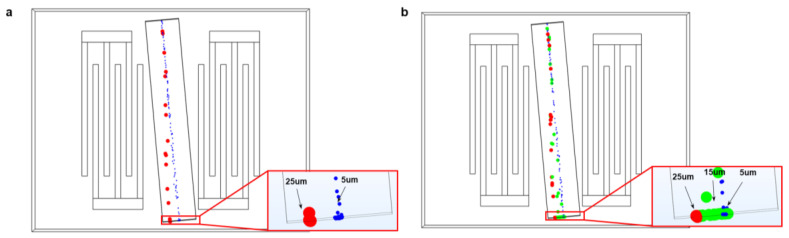
The particle trajectories of the single-stage SAW device with two different diameter particles (**a**) and with three different diameter particles (**b**).

Particles with diameters of 5 μm, 15 μm, and 25 μm are released at the inlet of the channel, which is located at the center of the inlet. The particle trajectories, under the coupling effect of the acoustic and flow fields, are shown in Figure 5. Since ARF is proportional to *a*^3^ (*a* is the particle diameter), the 15 μm and 25 μm particles are subject to a strong ARF. The 25 μm and 15 μm particles traverse multiple pressure nodes and antinodes due to a relatively large ARF. The 5 μm particles receive insufficient acoustic radiation and therefore move along the flow direction. The separation trajectory results of the 5 μm and 25 μm particles are shown in Figure 5a. It can be seen, in the outlet, that the separation between the 5 μm and 25 μm particles is effective. However, when three different diameter particles flow into the channel, such as 5 μm, 15 μm, and 25 μm, it is difficult to separate them totally. From Figure 5b, the 15 μm particles were mixed with the 5 μm and 25 μm particles. Thus, the device system should be optimized to separate multi-size particles.

### 3.3. Influence of the Slanted Angle

**Figure 6:**
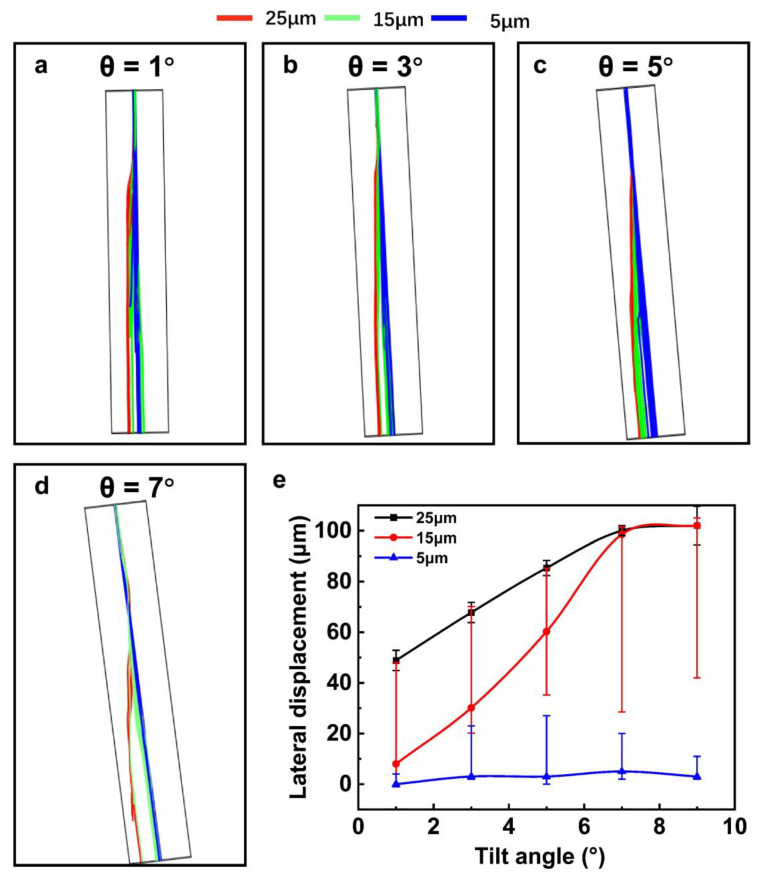
The particle trajectories under different slanted angles (**a**–**d**), the summary of the separation distance at different slanted angles (**e**).

To further improve the separation efficiency, we investigated the separation efficiency dependent on the slanted angle θ by numerical simulations. In this part, the acoustic pressure, the working frequency, and the flow rate were fixed at 0.75 MPa, 19.98 MHz, and 0.8 cm/s, respectively. We defined the center of the outlet of the channel as the zero point, and ΔZ represents the distance of the particle offset to the center position of the channel, which is the lateral displacement. The particle trajectories of the three different size particles under different slanted angles are shown in Figure 6. For the 5 μm particles, the ΔZ was nearly zero under different slanted angles θ, because of the insufficient ARF. For the 15 μm particles, the average ΔZ increased with the slanted angle θ from 1^o^ to 7^o^. However, the lateral distribution of 15 μm particles at the outlet is relatively large, and some of the particles were a mixture of 5 μm and 25 μm particles. For the particles with a diameter of 25 μm, their lateral separation displacement was increased with the angle θ changes from 1^o^ to 7^o^ and kept stable from 7° to 8°. 

From the above results and analysis, the separation characteristic is summarized in Figure 6e. With the angle increase, the relative separation distance between the 5 μm and 25 μm increased. However, it was still difficult to pick out the 15 μm particles by optimizing the slanted angle. With the angle shift from 1^o^ to 4^o^, the 15 μm particles were mixed both with 5 μm and 25 μm particles, and with the angle shift from 5^o^ to 8^o^, the 15 μm particles were mixed with 25 μm particles.

### 3.4. Influence of the Acoustic Pressure 

**Figure 7:**
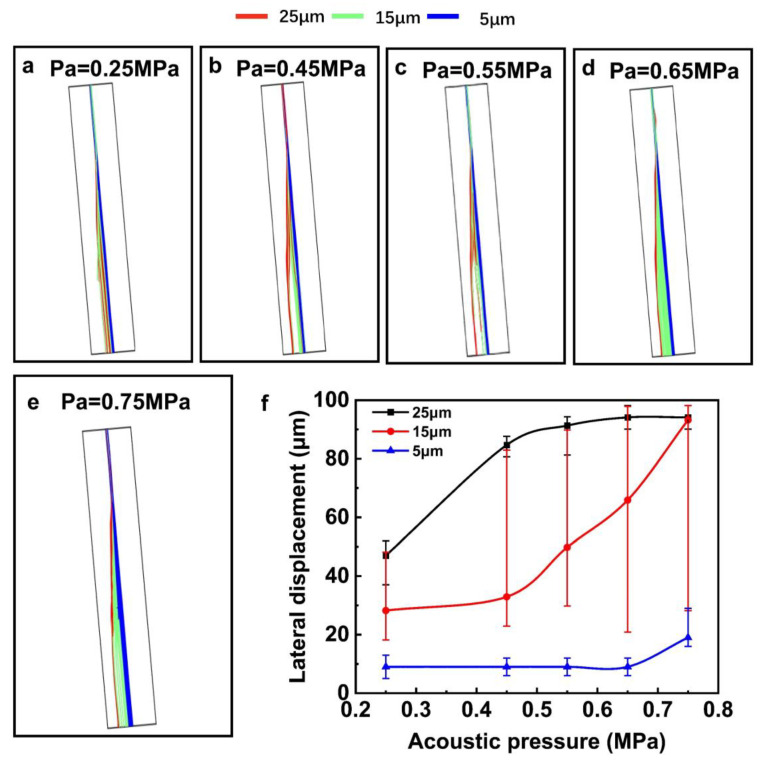
The particle trajectories under different acoustic pressures (**a**–**e**), the summary of the separation distance at different acoustic pressures (**f**).

The driving signal’s amplitude has a significant impact on the particles’ lateral movement, which determines the amplitude of acoustic pressure produced in the micro-channel. In this part, the slanted angle of the channel was set as 5°, while the flow rate of the liquid was fixed at 0.8 cm/s. The working frequency of the SAW device is 19.98 MHz. The particle trajectories at different acoustic pressures are shown in Figure 7a–e. With the increased acoustic pressure, the ARF received by the particle increases. At the pressure of 0.25 MPa, the particles of 25 μm showed a lateral displacement of 50 μm, while the 15 μm showed a displacement range of 20 μm to 50 μm, and the 5 μm particles exhibited no displacement. With the acoustic pressure increase to 0.45 MPa, the deflection of the 15 μm and 25 μm particle trajectories increases. However, although the lateral displacement of the 15 μm particles increases, its distribution is a little random, and some of the particles are mixed with 25 μm particles. For the particles with a diameter of 25 μm, when the acoustic pressure changed from 0.25 MPa to 0.55 MPa, the deflection increased from 45 μm to 90 μm. With the continuous increase in acoustic pressure from 0.55 MPa to 0.75 MPa, the deflection of the 25 μm particles was kept at ~90 μm. This is because the leftmost node pressure line in the channel was at the distance of 90 μm. For the particles of 15 μm diameter, when the acoustic pressure is 0.45 and 0.55 MPa, they were separated from the 5 μm particles. The summary of the separation results of the three different diameters is shown in Figure 7f, indicating that through optimizing the acoustic pressure, it was still difficult to separate the three different diameter particles with high efficiency as well. 

### 3.5. Effects of the SAW’s Resonant Frequency

**Figure 8:**
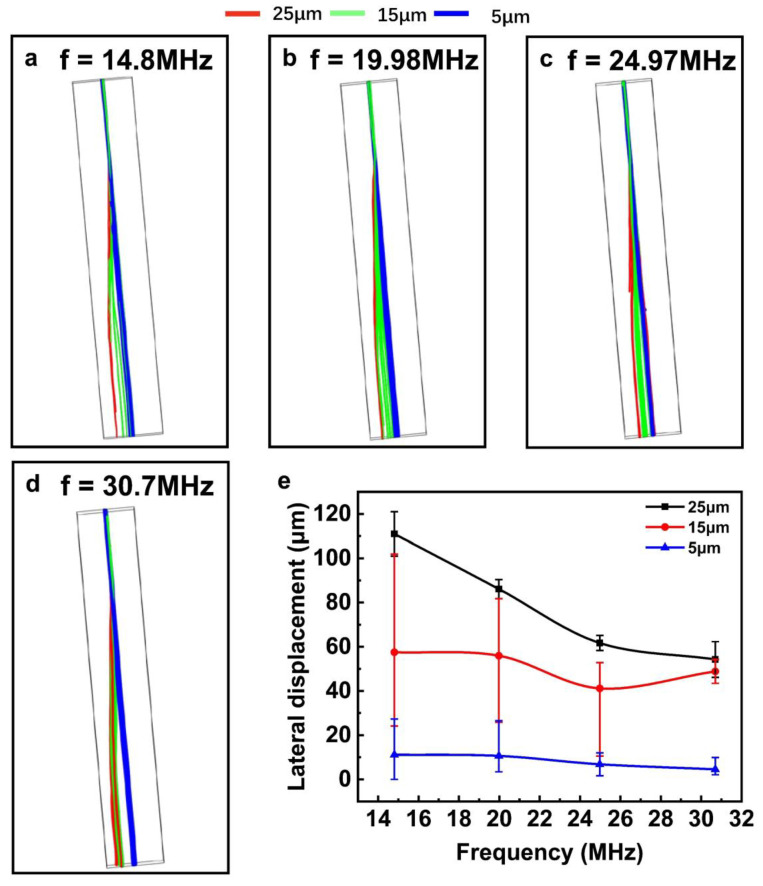
Flow trajectories of particles with different resonant frequencies (**a**–**d**) (14.8 MHz, 19.98 MHz, 24.975 MHz, 30.7 MHz); the lateral displacement summary of the particles under various driving frequencies (**e**).

The SAW device’s operating frequency affects the width of the acoustic pressure line in the channel, which is crucial for particle sorting. According to *f* = *c/λ*, the frequency can be adjusted by changing the width of the IDT fingers. The corresponding resonant frequencies of SAW devices with wavelengths of 270, 200, 160, and 130 μm are 14.8, 19.98, 24.975, and 30.7 MHz, respectively. As is shown in Figure 8, particles of 5 μm continue to move forward along the original direction at different frequencies without deflection, while particles of other sizes could produce lateral displacements at different operating frequencies. When the SAW device is at a lower frequency, only particles with a size of 25 μm are completely separated. This is because the energy density of acoustic waves is low at lower frequencies, which cannot cause lateral displacement of small particles. As the frequency continues to increase, the 15 μm particles are also deflected. Therefore, an increase in frequency is more effective for separating particles of smaller size, which is consistent with the existing research results [37]. A larger density of acoustic energy could be produced by increasing frequency, suitable for moving smaller particles.

### 3.6. The Particle Separation of Multistage SAW

To remove the restrictions of separating the particles with more than three different diameters with high efficiency, a multi-stage SAW device was proposed in this work. The structure of the SAW device is shown in Figure 1b and Figure 2b. The specific design parameters of multi-stage IDTs are shown in Table 3. 

**Figure 9:**
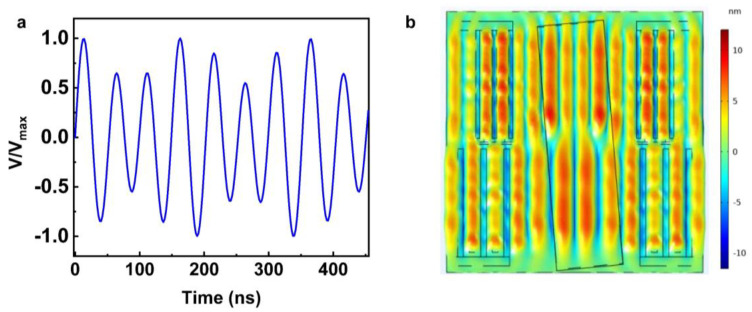
The modulated driving signal composed of two different frequencies with different amplitudes (**a**); the surface displacement generated by the multi-stage IDT driven by modulated signal (**b**).

The surface acoustic waves generated by the IDT must be continuous to allow the classification of particles of various sizes without losing the resolution. The integrated driving signal was shown in Figure 9a, the signals with two different frequencies were coupled together, and the amplitude of the two signals could be changed depending on the application. Through time-domain simulation studies, we can observe that continuous SSAWs are generated on the surface of the device. From Figure 9b, we can see the surface displacement generated by two electrodes with different wavelengths. In addition, the SSAW profile was related to the SAW wavelength. Therefore, the programable acoustic pressure could be generated by changing the wavelength of the IDT as well as the amplitude of the driving signal. We believe this technique can be applied in real applications for highly efficient separation of particle mixtures.

**Figure 10:**
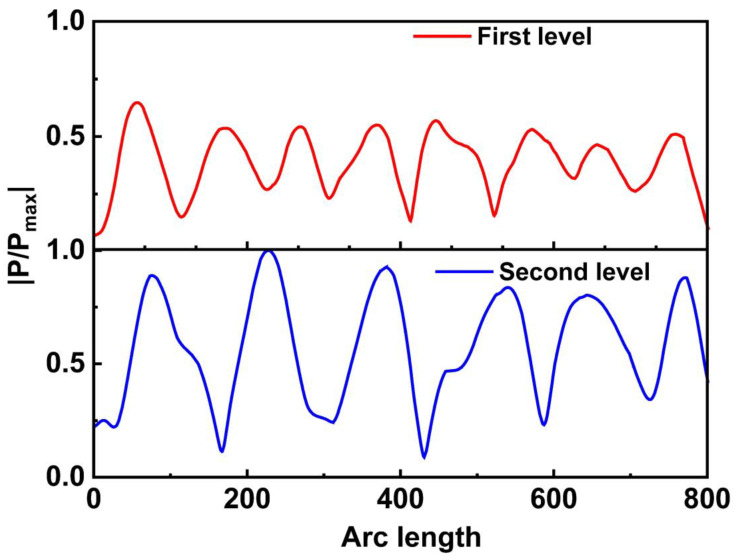
The normalized acoustic pressure generated by the multi-stage SAW device.

In theory, if different input voltages of the two frequencies were applied, the vibration amplitude and the acoustic pressure of the SAW would be different. Therefore, it is possible to determine the distribution of various acoustic pressure amplitudes by modifying the input amplitude. For the designed multi-stage sorting device, the normalized acoustic pressure is shown in Figure 10. The periods of the two-stage acoustic pressure correspond to the wavelength of the IDT, and the acoustic pressure amplitude is related to the driving signal’s amplitude. 

**Figure 11:**
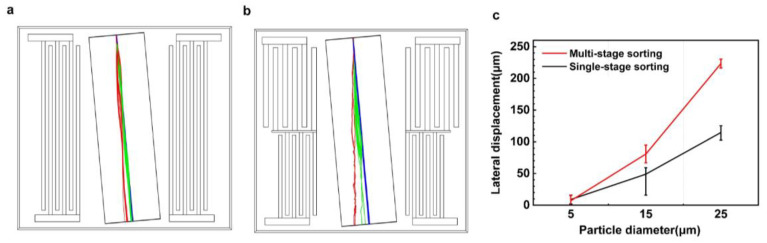
Comparison of the trajectories of sorting particles with three different particle sizes using a single-stage (**a**) and multi-stage sorting device (**b**); the lateral displacement summary of the particles under the single-stage and multi-stage SAW device (**c**).

Based on the results of single-stage separation, the separation of three different particle sizes achieved by multi-stage IDT with an acoustic pressure at 0.45 MPa in the first stage (top) and an acoustic pressure at 0.75 MPa in the second stage (bottom) is shown in Figure 11b, where the red lines represent the largest particles (25 μm), the green for medium-sized particles (15 μm), and the blue for the smallest (5 μm) particles. In the first stage, the ARF dominates the larger particles, which causes the particles to flow in the direction of the inclined node. In contrast, the drag force cancels most of the acoustic radiation force of the remaining particles, resulting in a small lateral displacement. When these particles enter the second sorting area, the acoustic radiation force continues to separate the largest particles farther away, and when the mixed particles move through the SAW, the medium particles were separated from the mixture by the high-frequency ARF, while the smallest particle still showed little displacement. Therefore, particles with a size of 25 µm can be discriminated in the first stage separation module and particles with a size of 15 µm can besorted in the second stage separation module. 

Figure 11c describes the particle separation distances of single-stage sorting and multi-stage sorting for three different particle sizes. It can be seen that the multi-stage sorting design was superior to single-stage sorting in terms of lateral displacement, and the particle trajectories do not overlap. The sorting efficiency of the multi-stage sorting for three different sizes of particles is noted as 99%, much higher than the 70% efficiency of single-stage sorting. 

For the rest of the particles mixed with different sizes, we can study the threshold value through single-stage sorting and efficiently separate the mixed particles through the modulation method of multi-stage sorting. This multi-stage sorting method can be used for the separation of various mixed cells in the blood. Human blood contains different types of cells, which is very important for their separation and purification. The proposed device may also be used to isolate cells from other hybrid organisms, and when our design is refined enough, it holds promise for isolating tiny viruses. The extension of the number of electrodes designed can increase the separation types.

## 4. Conclusions

In conclusion, an SSAW-based particle separation platform was proposed and simulated using the 3D FEM method. For the single-stage SAW device, we have demonstrated the influence of acoustic pressure, slanted angle, and working frequency (wavelength) on the separation distance of the particles. However, it was difficult to separate particles with three diameters with a single-stage device. To tackle this problem, an integrated multi-stage SAW device coupled with different wavelengths was proposed. To drive the multi-stage SAW device, a modulated signal composed of two different frequencies was generated. In addition, the amplitude of the two signals could be modulated as well. In the multi-stage SAW device, the corresponding different acoustic pressure was generated. In the first stage, the largest particle was separated first, and in the second stage, the medium-large particle was separated. The separation efficiency of the multi-stage device is noted as 99% for three different diameter particles. Moreover, the number of stages of the SAW device could be increased to three, four, or more, thus posing great potential in practical biomedical applications.

## Figures and Tables

**Table 1:** Model parameters.

Part $\Omega_{1}$				
	Single-stage		Multi-stage	
Substrate length	$L_{s}$	2500 μm	$L_{m}$	2500 μm
Substrate width	$W_{s}$	2810 μm	$W_{m}$	2810 μm
Length of IDT	$L_{a}$	1700 μm	$L_{b},L_{c}$	1100 μm
Width of IDT	$W_{a}$	50 μm	$W_{b},W_{c}$	50 μm, 70 μm
Wavelength	λ	200 μm	$\lambda_{1},\lambda_{2}$	200 μm, 280 μm
Part $\Omega_{2}$				
	Single-stage		Multi-stage	
Channel length	$L_{sc}$	2200 μm	$L_{mc}$	2350 μm
Channel width	$W_{sc}$	300 μm	$W_{mc}$	800 μm
Height	h	100 μm	h	100 μm

**Table 2:** Material parameters.

Water		
Density (kg/m^3^)	$\rho_{water}\;$	998
Speed of sound (m/s)	$c_{water}\;$	1481
Viscosity (Pa·s)	μ	1 × 10^−3^
Lithium niobate (LiNbO_3_)		
Density (kg/m^3^)	$\rho_{l}$	4700
Relative permittivity	$\left[\begin{array}{ccc} \varepsilon_{11} & 0 & 0\\ 0 & \varepsilon_{22} & 0\\ 0 & 0 & \varepsilon_{33} \end{array}\right]$	$\left[\begin{array}{ccc} 43.6 & 0 & 0\\ 0 & 43.6 & 0\\ 0 & 0 & 29.16 \end{array}\right]$
Elastic Constants (GPa)	$\left[\begin{array}{cccccc} C_{11} & C_{12} & C_{13} & C_{14} & 0 & 0\\ C_{21} & C_{22} & C_{23} & C_{24} & 0 & 0\\ C_{31} & C_{32} & C_{33} & 0 & 0 & 0\\ C_{41} & C_{42} & 0 & C_{44} & 0 & 0\\ 0 & 0 & 0 & 0 & C_{55} & C_{56}\\ 0 & 0 & 0 & 0 & C_{65} & C_{66} \end{array}\right]$	$\left[\begin{array}{cccccc} 202.8 & 52.9 & 74.9 & 8.9 & 0 & 0\\ 52.9 & 202.8 & 74.9 & -8.9 & 0 & 0\\ 74.9 & 74.9 & 243 & 0 & 0 & 0\\ 8.9 & -8.9 & 0 & 59.9 & 0 & 0\\ 0 & 0 & 0 & 0 & 59.9 & 8.9\\ 0 & 0 & 0 & 0 & 8.9 & 74.9 \end{array}\right]$
Piezoelectric Constants (C/m^2^)	$\left[\begin{array}{cccccc} 0 & 0 & 0 & 0 & e_{15} & e_{16}\\ e_{21} & e_{22} & 0 & e_{24} & 0 & 0\\ e_{31} & e_{32} & e_{33} & 0 & 0 & 0 \end{array}\right]$	$\left[\begin{array}{cccccc} 0 & 0 & 0 & 0 & 3.7 & -2.5\\ -2.5 & 2.5 & 0 & 3.7 & 0 & 0\\ 0.19 & 0.19 & 1.3 & 0 & 0 & 0 \end{array}\right]$
Polystyrene		
Density (kg/m^3^)	$\rho_{p}$	1050
Poisson’s ratio	$\sigma_{p}$	0.35
Speed of sound (m/s)	$c_{p}$	2350
IDT		
Density (kg/m^3^)	$\rho_{i}$	2700
Young modulus (GPa)	$E_{i}$	70
Relative permittivity	$\varepsilon_{i}$	1
Poisson’s ratio	$\sigma_{i}$	0.42
Polydimethylsiloxane (PDMS)		
Density (kg/m^3^)	$\rho_{PDMS}$	920
Speed of sound (m/s)	$c_{PDMS}$	1076.5

**Table 3:** Design parameters for the multi-stage electrodes.

	First-Stage IDT	Second-Stage IDT
Finger width	50 μm	70 μm
Finger spacing	50 μm	70 μm
Aperture length	1060 μm	1060 μm
IDT distance on both sides	1400 μm	1400 μm
Operating frequency	19.98MHz	14.8MHz
Wavelength	200 μm	280 μm

## Data Availability

Data are available upon request by contacting the corresponding author.
